# Response of Ruminal Microbiota–Host Gene Interaction to High-Altitude Environments in Tibetan Sheep

**DOI:** 10.3390/ijms232012430

**Published:** 2022-10-17

**Authors:** Yuzhu Sha, Yue Ren, Shengguo Zhao, Yanyu He, Xinyu Guo, Xiaoning Pu, Wenhao Li, Xiu Liu, Jiqing Wang, Shaobin Li

**Affiliations:** 1 College of Animal Science and Technology/Gansu Key Laboratory of Herbivorous Animal Biotechnology, Gansu Agricultural University, Lanzhou 730070, China; 2 Institute of Livestock Research, Tibet Academy of Agricultural and Animal Husbandry Sciences, Lhasa 850000, China; 3 School of Fundamental Sciences, Massey University, Palmerston North 4410, New Zealand; 4 Academy of Animal Science and Veterinary Medicine, Qinghai University, Xining 810000, China

**Keywords:** Tibetan sheep, microbiota, host, altitude, fermentation, immune

## Abstract

Altitude is the main external environmental pressure affecting the production performance of Tibetan sheep, and the adaptive evolution of many years has formed a certain response mechanism. However, there are few reports on the response of ruminal microbiota and host genomes of Tibetan sheep to high-altitude environments. Here, we conducted an integrated analysis of volatile fatty acids (VFAs), microbial diversity (16S rRNA), epithelial morphology, and epithelial transcriptome in the rumen of Tibetan sheep at different altitudes to understand the changes in ruminal microbiota–host interaction in response to high altitude. The differences in the nutritional quality of forage at different altitudes, especially the differences in fiber content (ADF/NDF), led to changes in rumen VFAs of Tibetan sheep, in which the A/P value (acetic acid/propionic acid) was significantly decreased (*p* < 0.05). In addition, the concentrations of IgA and IgG in Middle-altitude (MA) and High-altitude Tibetan sheep (HA) were significantly increased (*p* < 0.05), while the concentrations of IgM were significantly increased in MA (*p* < 0.05). Morphological results showed that the width of the rumen papilla and the thickness of the basal layer increased significantly in HA Tibetan sheep (*p* < 0.05). The 16S rRNA analysis found that the rumen microbial diversity of Tibetan sheep gradually decreased with increasing altitude, and there were some differences in phylum- and genus-level microbes at the three altitudes. RDA analysis found that the abundance of the *Rikenellaceae RC9 gut group* and the *Ruminococcaceae NK4A214 group* increased with altitudes. Furthermore, a functional analysis of the KEGG microbial database found the “lipid metabolism” function of HA Tibetan sheep to be significantly enriched. WGCNA revealed that five gene modules were enriched in “energy production and conversion”, “lipid transport and metabolism”, and “defense mechanisms”, and cooperated with microbiota to regulate rumen fermentation and epithelial immune barrier function, so as to improve the metabolism and immune level of Tibetan sheep at high altitude.

## 1. Introduction

The rumen of ruminants is a complex microbial ecosystem that evolved and developed approximately 35–40 million years ago [1]. It is part of the complex digestive system of ruminants and it is rich in microorganisms such as bacteria, protozoa, and fungi. Ruminants carry out specific processes involved in energy and lipid metabolism via the microbial degradation of the structural carbohydrates (cellulose, hemicellulose, and lignin) in forage [2]. The metabolite volatile fatty acids (VFAs) produced can stimulate the activation of the sympathetic nervous system and promote energy consumption [3], regulate rumen epithelial growth, hormone secretion, and the immune process [4,5]. In addition to fermenting feed, the ruminal microbiota plays a certain role in host immune regulation [6], disease prevention [7], energy balance [8], and physiological development [9]. As an environmental factor, microbiota interact with host genes to influence phenotype [10,11]. Over the past few decades, a large number of studies have shown that microbiota can improve the host’s ability to survive and reproduce in specific environments, including the regulation of morphological development, disease resistance, physiology, and adaptability [12]. Our previous study also found that plateau ruminants regulate nutrient absorption and barrier function through the interaction of rumen microbiota with VFAs and host genes under cold-season nutrient stress conditions [13]. Other studies have found that both the host’s genetic structure and rumen microbiota can affect the host’s metabolic phenotype, and the evaluation of the host’s phenotypic traits using its microbiota is even more accurate than evaluating them according to the host’s genetics [14]. The rumen epithelium serves as a place where the host and microbiota interact, affects the net nutrient utilization by the whole body, and is also an important physical barrier [15]. Therefore, there is an association between the host and its rumen microbes, and this association is used to coordinate the regulation of host metabolic functions.

Tibetan sheep have been grazing and living on the plateau for generations, and this is affected by many factors, such as severe cold, low oxygen, strong ultraviolet ray, nutrient deficiency in the cold season, etc. At present, studies on plateau adaptation mainly focus on blood physiological and biochemical indicators, morphology, and genome, and only a few studies focus on the microbiota coevolving with the host [16]. Altitude is the main external environmental pressure affecting the production performance of Tibetan sheep. At high altitude, the rumen microbiota plays an important role, and its metabolites interact with the host [17]. Plateau animals adapt to high-altitude environments by increasing their energy intake along with their beneficial metabolic mechanisms. Plateau mammals have a faster metabolic rate and have to consume more oxygen and energy for normal survival [18]. In this process, as an organ for feed digestion and energy conversion, the rumen plays a considerable role. Studies have found that microbes living in the Gastrointestinal (GI) play a crucial role in host adaptation [19,20,21]. In the Qinghai–Tibet Plateau, Tibetan studies on humans and animals have found that there are differences between human and animal gut microbiota in high-altitude and low-altitude areas, which further indicates that unique ruminal microbiota and its functions might be associated with plateau adaptability [22,23]; therefore, the plateau environment pressure can lead to changes in the abundance and composition of the ruminal microbiota [24]. In addition, Yan Ma’s study found that the intestinal microbiome composition and function of high-altitude herbivores are basically similar, suggesting that the intestinal microbiota may be related to the plateau environment [25]. Changes in altitude also affect the composition and structure of rumen microbiota, and then affect the structure and function of rumen tissues [26]. These studies indicate that the host and the environment both affect the microbiota, which further influences the host phenotype. Host genes and microbiota play an important role in host co-development [27,28], and the host and GI microbiota can respond to external environmental pressures [29,30]. Thus, this study hypothesized that the ruminal microbiota–host gene interaction of Tibetan sheep can respond to different altitude environments. We comprehensively analyzed the rumen VFAs levels, the microbiota, the rumen epithelial morphology, and the transcriptome interactions of Tibetan sheep at different altitudes, aiming to explore and reveal the following issues: (a) Differences in rumen microbial fermentation metabolism (VFAs) of Tibetan sheep at different altitudes; (b) Morphological changes in the rumen epithelium of Tibetan sheep under pressure at high altitude; (c) Regulation of fermentation metabolism and immune barrier by microbiota–host gene interaction in the rumen of Tibetan sheep at high altitude.

## 2. Results

### 2.1. Forage Species and Nutrient Composition at Different Altitudes

**Figure 1:**
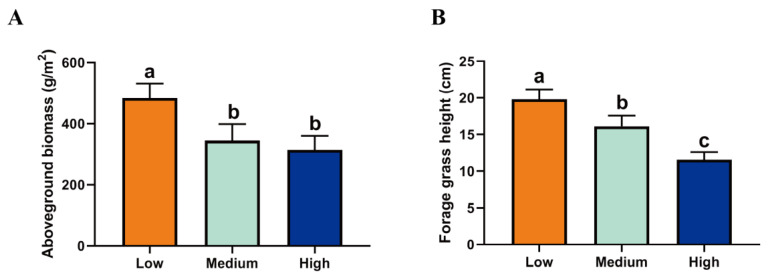
Forage growth at different altitudes: (**A**) Aboveground biomass; (**B**) Forage grass height. Note: Different lowercase letters above the column indicate significant differences, *p* < 0.05.

The aboveground biomass and height of forage on the Qinghai–Tibet Plateau decreased with increasing altitude (Figure 1). *Poa poophagorum Bor*, *Gramineae, Kobresia pusilla Ivan*, *Potentilla anserina* L., and *Geranium platyanthum Duthie* are the dominant forage at LA; *Poa pratensis* L., *Elymus nutans Griseb, Agropyron cristatum* (L.) *Gaertn* and *Stipa aliena Keng* are the dominant forage at MA; and *Poa annua* L., *Oxytropis latibracteata Jurtzev, Medicago archiducis-nicolai G. Sirjaev, Potentilla bifurca*, and *Adonis caerulea* are the dominant forage at HA. Due to altitude and forage species (Table 1), forage fiber content (NDF, ADF, and HCEL) in the MA group was significantly higher than that of the other two groups (*p* < 0.01), while NFC content was the lowest. The contents of CP, Ash, Ca, and P in HA were significantly higher than those of the other groups (*p* < 0.01).

### 2.2. Serum Immune Levels of Tibetan Sheep at Different Altitudes

**Figure 2:**
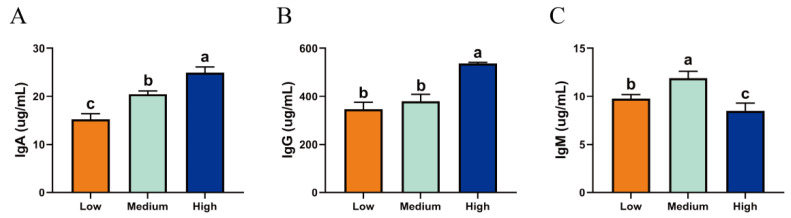
Serum immune indices of Tibetan sheep at different altitudes: (**A**) IgA; (**B**) IgG; (**C**) IgM. Note: Different lowercase letters above the column indicate significant differences, *p* < 0.05.

There were differences in the immunoglobulin contents in the serum of Tibetan sheep at different altitudes (Figure 2). The IgA contents of Tibetan sheep at LA were significantly lower than those at MA and HA (*p* < 0.05), and the contents of IgA and IgG in the serum of HA sheep were the highest, and the content of IgM at HA was significantly lower than that at other altitudes (*p* < 0.05).

### 2.3. Characteristics of Rumen VFAs and Epithelial Morphology

As shown in Table 2, there were certain differences in rumen VFAs at different altitudes. The VFAs content at MA was significantly higher than that at LA and HA (*p* < 0.05), and the VFAs content at HA was the lowest. The contents of acetic acid and butyric acid at HA were significantly lower than those in the other altitudes (*p* < 0.05), while isobutyric acid significantly decreased at HA (*p* < 0.05). In addition, valeric acid was not significantly different at the three altitudes (*p* > 0.05).

**Figure 3:**
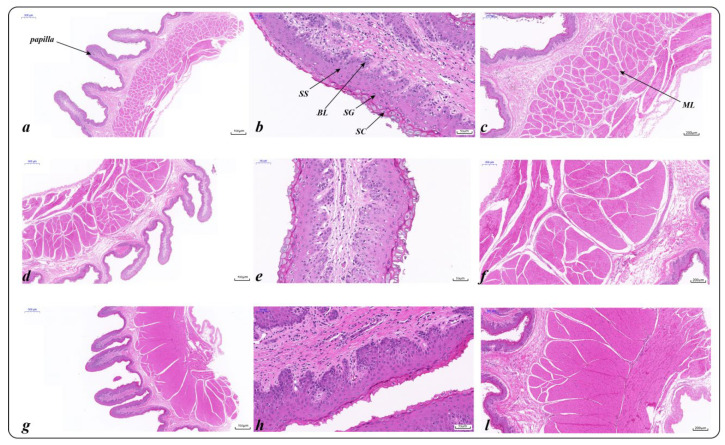
Ruminal epithelial structures of Tibetan sheep at different altitudes: LA: (**a**–**c**); MA: (**d**–**f**); HA: (**g**–**l**); SC: stratum corneum; SG: stratum granulosum; SS: stratum spinosum; BL: basal layer; ML: muscular layer.

The rumen epithelial development and structural characteristics of Tibetan sheep at different altitudes were different (Figure 3). The rumen muscle of MA was significantly thicker than that of the other altitudes (*p* < 0.05), and the papilla width of the HA group was significantly larger than that of other altitudes (*p* < 0.05). The rumen stratum corneum at MA was significantly thicker than that in the other altitudes (*p* < 0.05), the thickness of the stratum granulosum decreased significantly at HA (*p* < 0.05), and the basal layer thickness increased significantly at HA (Table S1).

### 2.4. Microbial Diversity of Tibetan Sheep at Different Altitudes

**Figure 4:**
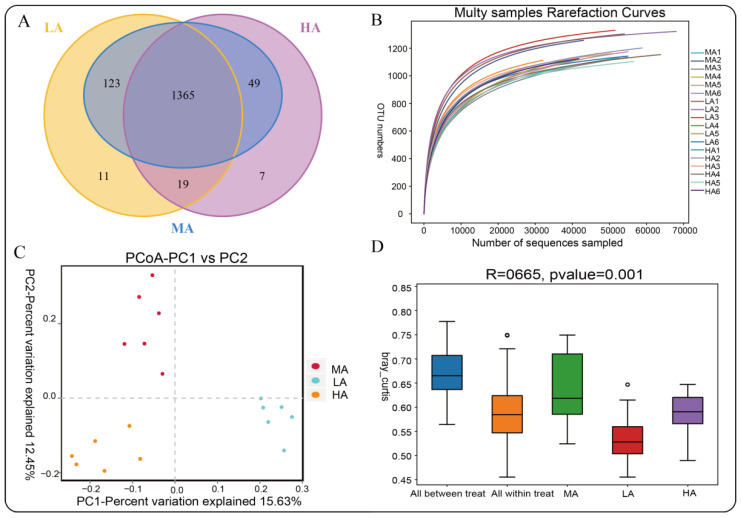
Analysis of the microbial diversity of Tibetan sheep at different altitudes: (**A**) OTU-Venn diagram analysis; (**B**) Rarefaction curve analysis; (**C**) PCoA analysis; (**D**) Anosim boxplot.

A total of 1,440,989 pairs of Reads were obtained by rumen fluid sequencing, and 1,431,444 Clean Reads were generated after paired-end Reads quality control and splicing, with an average of 79,525 Clean Reads generated per sample and an average sequence length of 418 bp. Cluster analysis obtained 1574 OTUs, among which there are 11 unique OTUs in the LA group and 7 unique OTUs in the HA group (Figure 4A). Analysis of the rarefaction curves found that the curve flattens out at 50,000 reads, indicating that sequencing coverage had saturated and was ready for subsequent analysis (Figure 4B). PcoA/Anosim analysis found that there were differences among the three altitudes, and the differences among the groups were greater than the differences within the groups (Figure 4C,D). Alpha diversity analysis showed that the Shannon index of LA Tibetan sheep was higher than that of MA and HA, indicating that the rumen microbial diversity of LA Tibetan sheep was higher. ACE indexes show that the rumen microbial richness of MA is larger than LA and HA, and Coverage is above 99% (Table 3).

### 2.5. Microbial Composition and Abundance of Tibetan Sheep at Different Altitudes

**Figure 5:**
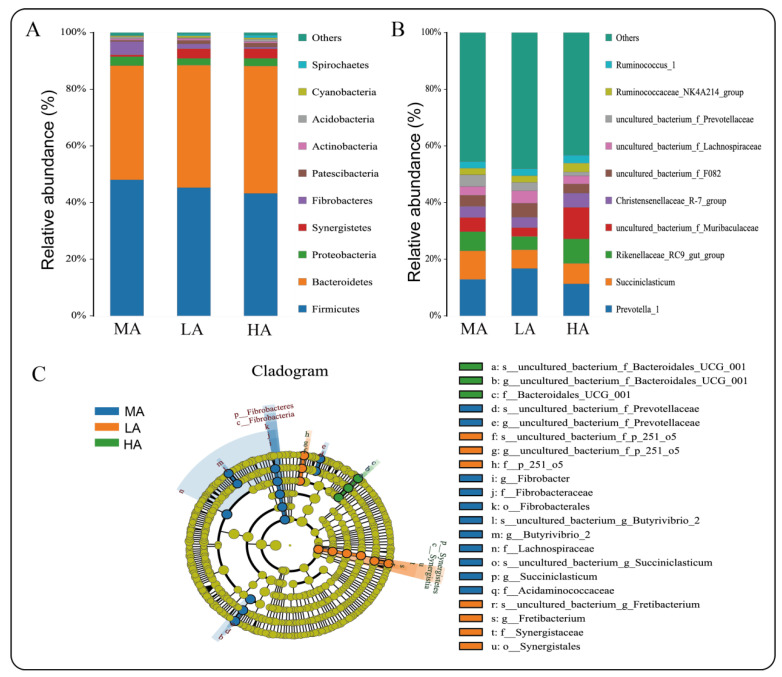
Analysis of the microbial composition and abundance of Tibetan sheep at different altitudes: (**A**) Relative abundance of species at phylum level; (**B**) Relative abundance of species at genus level; (**C**) LEfSe analysis.

A total of 19 phyla, 36 classes, 73 orders, 116 families, 243 genera, and 280 species were detected from 18 samples. At the phylum level (Figure 5A), Firmicutes and Bacteroidetes were the dominant phyla at three altitudes, accounting for more than 88% of the total. The abundance of Firmicutes at MA and LA was greater than that of Bacteroidetes, while at HA, the abundance of Bacteroidetes was greater than that of Firmicutes. Second, Proteobacteria, Synergistetes, Fibrobacteres, and other bacteria had higher abundances. The Metastats analysis showed that there were seven different microbiota at the phylum level (Supplementary Materials Data Sheet S1), among which Tenericutes and Gemmatimonadetes were found in the LA and MA comparisons. There were five different bacterial phyla (Acidobacteriota, Gemmatimonadota, Tenericutes, Rokubacteria, and Fibrobacterota) between HA and LA, whereas there were no differences found between MA and HA (Supplementary Materials Data Sheet S1). At the genus level (Figure 5B), an analysis of different species showed that there were 10 different genera between LA and MA, 6 different genera between MA and HA, and 86 different genera between HA and LA (Supplementary Materials Data Sheet S1). A LEfSe analysis of samples between groups showed 25 different biomarkers, and that the abundance of *Uncultured_bacterium_f_Bacteroidales_UCG-001* in HA Tibetan sheep was significantly higher than that in the other groups (*p* < 0.05, Figure 5C).

**Figure 6:**
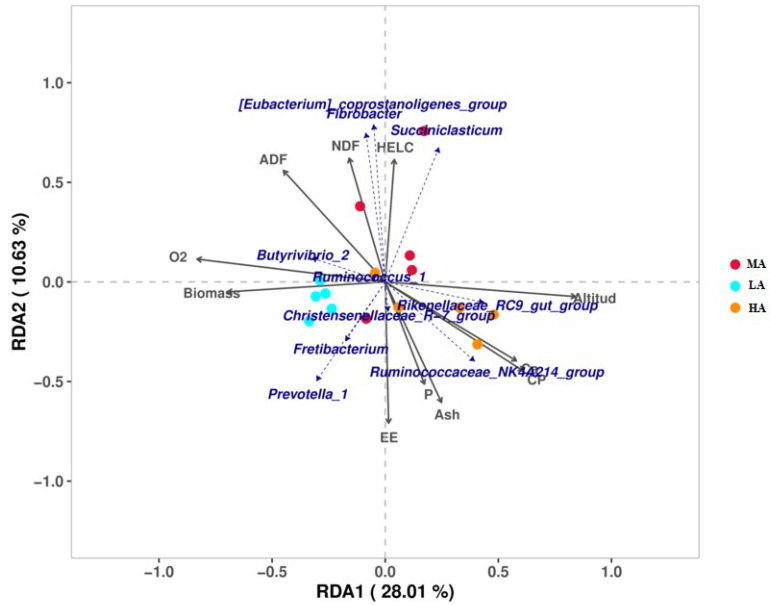
RDA analysis of environmental factors and microbiota. Note: The solid arrows represent environmental factors and the dashed arrows represent microorganisms, and the magnitude of the angle between them represents the correlation.

RDA analysis found that there was a correlation between altitude, O_2_, biomass, and microbiota (Figure 6). The abundance of *Rikenellaceae_RC9_gut_group* was significantly positively correlated with altitude (*p* < 0.05), and it increased with increasing altitude. In addition, altitude also had a certain correlation with *Ruminococcaceae_NK4A214_group*, while the abundance of *Butyrivibrio_2* decreased with decreasing environmental oxygen content. Second, it also had a certain correlation with *Ruminococcaceae_NK4A214_group*, while the *Butyrivibrio_2* abundance decreased with decreasing environmental oxygen content. The abundance of *Ruminococcus_1* also had a certain correlation with O_2_ content, which increased with the decrease in O_2_ content.

### 2.6. Microbial Gene Function

**Figure 7:**
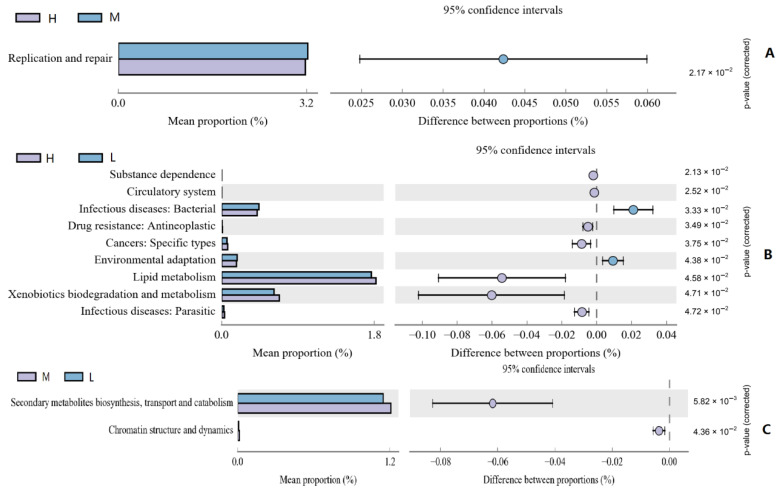
Microbial function analysis: (**A**,**B**) KEGG functional pathway; (**C**) COG functional pathway.

A total of 46 KEGG gene families and 25 COG gene families were identified by 16S rRNA gene function prediction. In the KEGG gene family, the replication and repair functions of HA and MA were significantly different (*p* < 0.05, Figure 7A), and nine different functions were found between HA and LA (Figure 7B), among which lipid metabolism accounted for the largest proportion, and HA was significantly higher than LA (*p* < 0.05); the xenobiotics biodegradation and metabolism function of HA was significantly increased, and the infectious diseases (Bacterial) function of HA was significantly lower than that of LA (*p* < 0.05). Among the functions of the COG gene family (Figure 7C), there are only two differential gene functions between MA and LA, namely, secondary metabolite biosynthesis, transport, and catabolism, and chromatin structure and dynamics, both of which show that MA is higher than LA (*p* < 0.05).

### 2.7. Microbiota–Host Gene Interactions Influence Rumen Fermentation and the Epithelial Barrier

#### 2.7.1. WGCNA of Host Transcriptome and Phenotypic Traits

**Figure 8:**
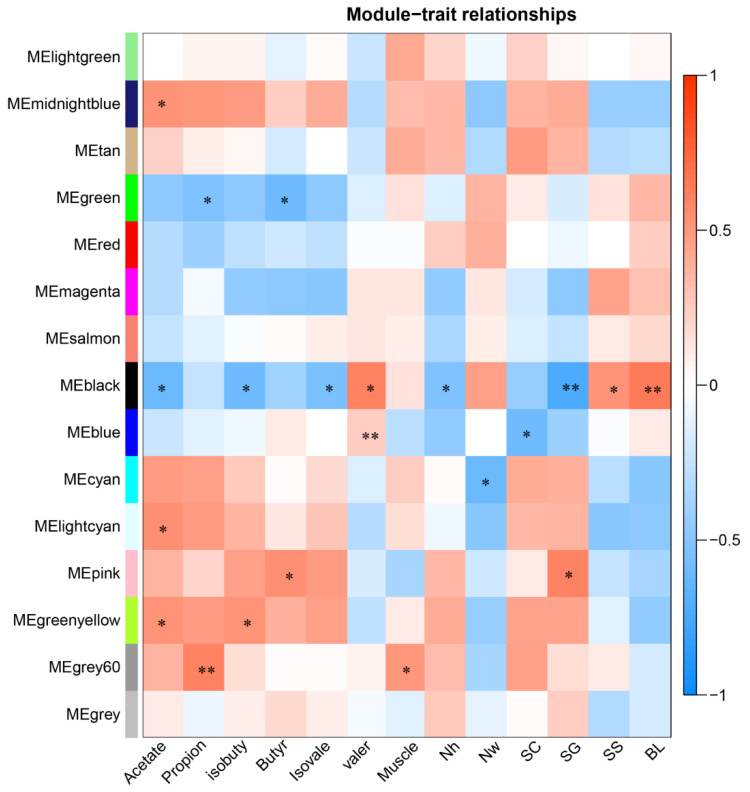
Heat map of correlation between gene modules and phenotypic traits. * *p* < 0.05, ** *p* < 0.01.

**Figure 9:**
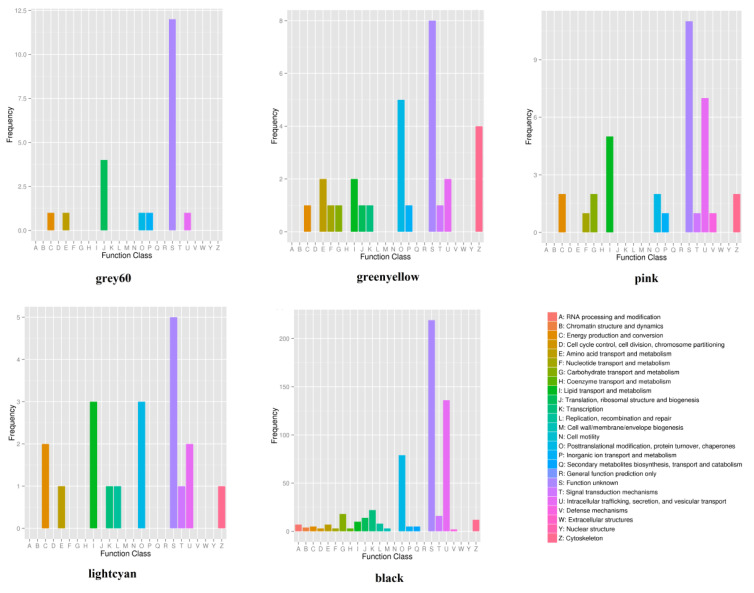
Functional enrichment analysis of gene modules.

Transcriptome analysis revealed that a total of 27,916 genes were expressed in the rumen tissues of Tibetan sheep at different altitudes. Weighted gene coexpression network analysis (WGCNA) showed that the rumen-expressed genes of Tibetan sheep at different altitudes were divided into 15 gene modules (Supplementary Materials Data Sheet S2), and these gene modules were associated with phenotypic traits related to rumen physiology (VFAs, rumen epithelial structure) to varying degrees (Figure 8). MEgray60, MEgreenyellow, MEpink, MElightcyan, and MEblack were strongly correlated with rumen fermentation (VFAs), while MEblack and MEpink were correlated with the rumen epithelial barrier. The MEgreenyellow and MElightcyan modules were positively correlated with acetic acid formation (*p* < 0.05). The coexpressed host genes (*gene CLDN23*, *gene LOC114110557*, etc.) (Figure S1) in these modules are enriched in “energy production and conversion”, “amino acid transport and metabolism”, “lipid transport and metabolism” and “intracellular trafficking, secretion, and vesicular transport” (Figure 9). The coexpressed genes in the MEgray60 module (*gene-LOC114109518*) were significantly positively correlated with propionic acid and were mainly enriched in “translation, ribosomal structure, and biogenesis”, “energy production and conversion”, and “amino acid transport and metabolism”. The MEPink module was significantly positively correlated with butyric acid (*p* < 0.05), and the coexpressed genes (*WDR66*, *gene-LOC105611550*, and *gene-SLC6A4*) were mainly enriched in “lipid transport and metabolism”, “carbohydrate transport and metabolism”, “energy production and conversion”, and “defense mechanisms” (Figure 9).

The co-expressed genes in the MEblack module were significantly negatively correlated with the stratum granulosum (*p* < 0.01), and was positively correlated with stratum spinosum and basal layer (*p* < 0.05), in which some genes primarily related to the body’s immune barrier (*CLDN7*, *ILs*, etc.) were enriched in “intracellular trafficking, secretion, and vesicular transport”, “posttranslational modification, protein turnover, and chaperones”, “carbohydrate transport and metabolism”, and “defense mechanisms”. MEPink was positively correlated with the stratum granulosum (*p* < 0.05), and *SLC6A4* was enriched in “inorganic transport and metabolism” (Figure 9).

#### 2.7.2. Interaction between Host Genes and Microbiota

**Figure 10:**
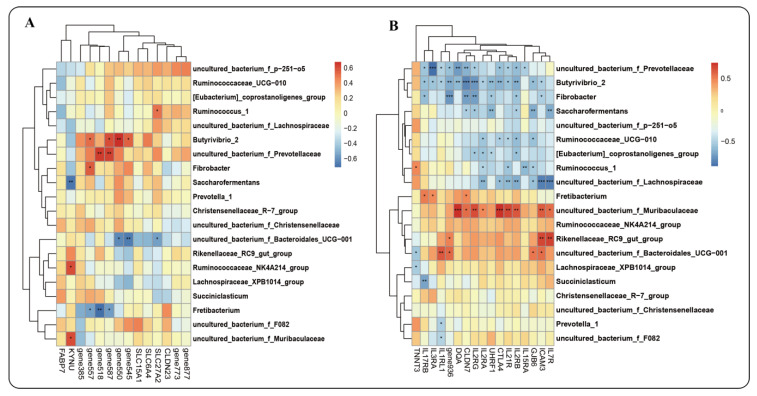
Microbiota–host gene interaction heatmap: (**A**) Heatmap of fermentation function; (**B**) Heatmap of barrier-related functions. * *p* < 0.05, ** *p* < 0.01, *** *p* < 0.001. Note: gene-877 (gene-LOC114117877); gene773 (gene-LOC114117773); gene545 (gene-LOC101119545); gene550 (gene-LOC105611550); gene587 (gene-LOC101117587); gene518 (gene-LOC114109518); gene557 (gene-LOC114110557); gene385 (gene-LOC114115385); gene 936 (gene-LOC114117936).

Based on the functional analysis of the above modules, the genes in these modules were further analyzed for their association with microbiota. As shown in Figure 10, genes related to fermentation function were significantly positively correlated with *Butyrivibrio_2, uncultured_Bacterium_f_Prevotellaceae, Fibrobacter, Ruminococcaceae_NK4A214_group*, and other bacteria (*p* < 0.05), but negatively correlated with *Saccharofermentans, Fretibacterium,* and *Uncultured_bacterium_f_ Bacteroidales_UCG−001* (*p* < 0.05). Moreover, epithelial barrier genes were correlated with most rumen bacteria, such as *Butyrivibrio_2, Fibrobacter, Saccharofermentans, Ruminococcaceae_UCG−010, Ruminococcus_1*, etc., showing a significant negative correlation (*p* < 0.05). *Rikenellaceae_RC9_gut_group, uncultured_bacterium_f_Muribaculaceae,* and *uncultured_bacterium_f_Bacteroidales_UCG−001* showed a significant positive correlation (*p* < 0.05), most of which belong to the IL family (Figure S2) and were mainly enriched in “defense mechanisms”, “intracellular trafficking”, “fluctuation and vesicular transport”, and other functions. The secondary function of microbiota is similar to the enrichment function of the host genes, which are enriched in “lipid metabolism”, “carbohydrate transport and metabolism”, and “defense mechanisms”.

## 3. Discussion

Currently, there is no study on rumen microbiota and host gene interaction in response to environmental pressure at different altitudes in Tibetan sheep. Tibetan sheep remain under natural grazing conditions year round, with natural forage being their main source of nutrition [31]. In this study, altitude affects the growth of forage, and with the increase in altitude, the aboveground biomass and height decrease, thus affecting the nutrient supply of grazing Tibetan sheep [32]. The microbial community in the rumen ferments forage into VFAs, which are then absorbed by the epithelium as energy materials [33]. According to previous studies, cellulose is an important factor affecting the content of VFAs [34,35]. In this study, since *Poaceae Barnhart* and *Poa annua L.* are the dominant forage grasses in MA, the content of cellulose (NDF/ADF/HCEL) is higher, and forage quality decreased, but the rumen VFAs content of Tibetan sheep in MA is significantly higher than that of Tibetan sheep at low altitudes. The reason for this result may be that grazing Tibetan sheep eat a large amount of forage or improve the fermentation function, thus increasing the content of fermentation metabolites VFAs in response to the extreme plateau environment, which also needs to be further verified. However, this result is consistent with our previous results [13], indicating that the higher the forage fiber in the cold season, the higher the rumen VFAs. The lower the ratio of acetic acid to propionic acid (A/P), the higher the energy utilization of forage [34]. Therefore, Tibetan sheep in the HA area showed stronger energy use efficiency, so as to maintain normal production performance. This is similar to previous studies, which show that animals can respond to different feeding conditions and stages, so as to maintain a normal balance of energy and nutrient supply and demand to adapt to production levels [36]. In addition, the forage grass in the HA area is mainly composed of legumes rich in protein, Ca, P, and other mineral elements, such as *Oxytropis latibracteata Jurtzev* and *Medicago archiducis-nicolai G. Sirjaev*. Thus, the nutrient value of forage increases and the fiber content decreases in the high-altitude area, and the body reduces the energy consumed in the fiber fermentation process. The rumen epithelium is an important part of nutrient absorption and the immune barrier [37]. Due to the influence of forage fiber content, the rumen muscle layer and stratum corneum of Tibetan sheep in MA are significantly thickened, thus responding to the special plateau environment [38]. In addition, the rumen papillary width of Tibetan sheep at the HA area was significantly increased, which expands the contact area of chyme, and ensures that more VFAs are absorbed and transported into the body, which also proves the reason for the lower VFA content at HA. The thickness of the rumen basal layer of Tibetan sheep in the HA area is also increased, which may increase the energy metabolic rate of the epithelial layer [39], so as to cope with the high-altitude environment. In addition, animal species, feeding time, and feeding conditions have certain effects on body homeostasis and physiology [40], among which humoral immune indicators IgA and IgG can reflect the immune status of the body [41,42]. This study found that the immunity of Tibetan sheep at high altitude was improved compared with that of Tibetan sheep at low altitude of 2500 m, and it indicates that Tibetan sheep are more suitable for living in an environment above 3000 m.

As the “second genome” of animals, the digestive tract microbiota plays an important role in their adaptation to the plateau environment [43]. This study found that the Shannon index decreased with the increase in altitude, which resulted in a decrease in rumen microbiota diversity. At the phylum level, Bacteroides increase with the increase in altitude, we also found that the higher protein content of forage at high altitudes, and therefore the higher abundance of Bacteroidetes, played a role in protein degradation [44], thus providing energy for the body. Fibrobacteres are believed to be the main bacterial degraders of lignocellulosic substances in the rumen of herbivores [45], and it was enriched in the rumen of Tibetan sheep at high altitude in this study. In RDA analysis, the genus-level species *Rikenellaceae_RC9_gut_group* and *Ruminococcaceae_NK4A214_group* have certain correlations with altitude. Studies have shown that *Rikenellaceae_RC9_gut_group* plays a role in the degradation of plant-derived polysaccharides [46], thereby degrading polysaccharides in plateau forage, and *Ruminococcaceae_NK4A214_Group* has a significant correlation with vitamin A [47], which is related to vitamin metabolism. These results suggest that Tibetan sheep at HA can adjust their energy content and vitamin metabolism through microbial changes to cope with extreme environments. In addition, with the increase in altitude, O_2_ content in the environment increases, and *Butyrivibrio_2* abundance in the rumen of Tibetan sheep in this study also decreases. Studies have found that the number of *Butyrivibrio* is positively correlated with the number of methanogens [48]. These results indicate that the abundance of methanogens in high-altitude Tibetan sheep is also reduced, which leads to a decrease in methane emissions, which is consistent with the characteristics of low-methane emissions in high-altitude animals, thus reducing energy waste. The KEGG function prediction of microbiota shows that the lipid metabolism of high-altitude Tibetan sheep increased significantly, while the xenobiotic biodegradation and metabolism decreased, thus suggesting that VFAs produced by HA Tibetan sheep may be involved in lipid metabolism and provide energy for the body [1].

**Figure 11:**
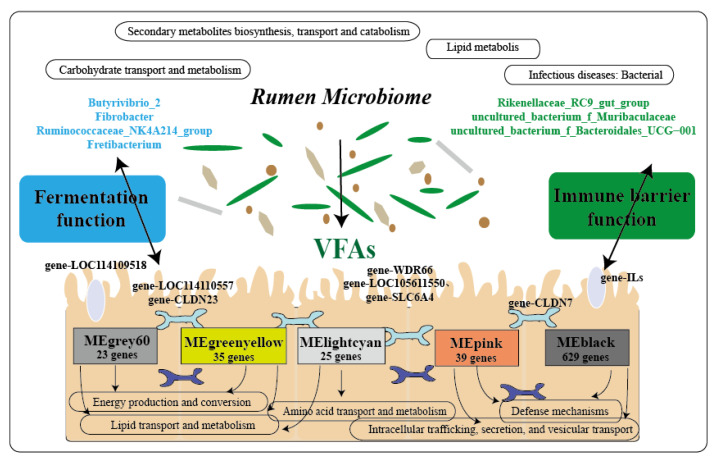
Model diagram of rumen microbial–host gene interaction regulation. Note: The upper part of the figure shows the secondary functional pathways of microorganisms, and the lower part shows the functional pathways enriched in epithelial module genes. On the left are the microbial flora and genes related to fermentation function, and on the right are the microbial flora and genes related to the immune barrier function.

To further explore the response of ruminal microbiota–host gene interaction to high altitude environment in Tibetan sheep. We conducted a comprehensive analysis of the host transcriptome, microbiome, and phenotypic traits, and it was found that rumen fermentation and immune barrier function play a role in response to the plateau environment in Tibetan sheep (Figure 11). It was found that the module genes related to VFAs were mainly enriched in “energy production and conversion”, “amino acid transport and metabolism”, “lipid transport and metabolism”, and “carbohydrate transport and metabolism”, so as to ensure normal fermentation metabolism at high altitude. In this study, with increasing altitude, the expression of *CLDN23* and *WDR66* genes decreased, which increased the absorption efficiency of the rumen epithelium [49,50,51], transported and metabolized more acetic acid and butyric acids, participated in the ketogenic process of the rumen epithelium [52], ensured the normal energy demand, and maintained the homeostasis of microbiota [53]. There was a correlation between the co-expressed genes (*ILs,* etc.) under the MEblack module and the rumen epithelial barrier. *IL* gene families were mainly enriched in “defense mechanisms”, “intracellular trafficking, secretion, and vesicular transport”, and “posttranslational modification, protein turnover, chaperones”, thus participating in the process of epithelial immune barrier function. It can directly recognize bacterial cell wall components as part of the gastrointestinal (GI) immune system and improve the body’s immunity [54,55]. The MEPink module gene *SLC6A4* was enriched in “inorganic transport and metabolism” and controlled the transport and metabolism of organic ions through the rumen epithelial barrier. With increasing altitude, the expression of *SLC6A4* decreased and then participated in the transport and metabolism of organic ions. Further correlation analysis found that the genes involved in fermentation metabolism and the immune barrier are related to rumen microbiota, and these rumen epithelial transcription genes are regulated by rumen microbes [56,57]. In this study, the cellulose-degrading bacteria *Butyrivibrio_2* and *Fibrobacter* [58] showed a significant positive correlation with fermentation-related genes (*gene-loc114110557, gene-loc105611550*). *Gene-LOC114110557* is a fatty acid-binding protein, and it has been reported that fatty acid-binding protein 7 (*FABP7*) is involved in fatty acid uptake, transport, and metabolism [59]. These results indicate that cellulolytic bacteria produced VFAs through forage fermentation, which were then controlled by rumen epithelium-related genes for absorption, transport, and metabolism (Figure 11). In HA regions, the abundance of *Butyrivibrio_2* was decreased (energy intake was reduced), while genes related to immune barriers were highly expressed, such as TJ membrane protein family member (*CLDN7*) [60], immune factor receptor (*IL2RG*) [61], and extracellular regulator of T-cell response (*CTLA4*) [62]. High-altitude Tibetan sheep regulate the immunity of the organism by regulating the expression of these genes, and we speculate that *Butyrivibrio_2* is also involved in regulation by decreasing abundance. Furthermore, the *Uncultured_Bacterium_f_Muribaculaceae* had a significant positive correlation with immune barrier-related genes, and some studies found that the abundance of *Muribaculaceae* was related to immunity [63]. Therefore, it is speculated that there is an inevitable relationship between the expression of these immune genes and the abundance of bacteria, which may synergistically regulate the body’s immunity. In addition, we can see that the host gene functional pathway was similar to the secondary functional pathway of microbial enrichment (Figure 11), which may be involved in regulating the metabolic barrier of the body. Based on the above analysis, rumen microbial–host gene interactions may respond to environmental pressure at different altitudes in Tibetan sheep.

## 4. Materials and Methods

### 4.1. Experimental Design and Location

**Figure 12:**
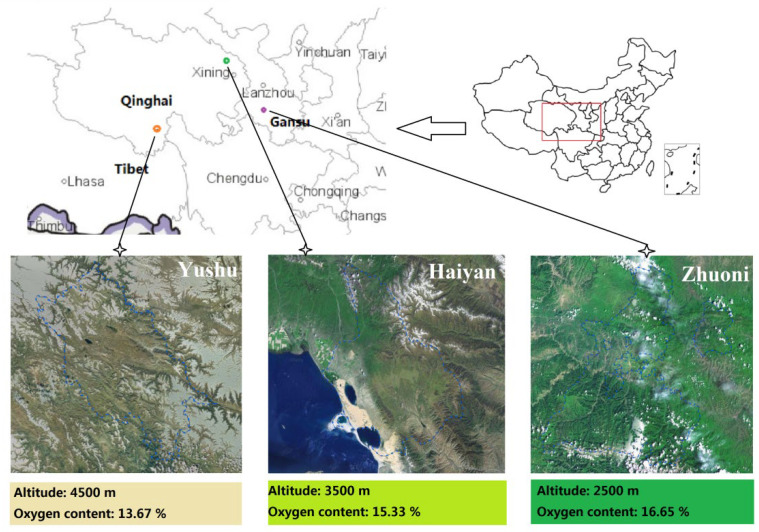
Qinghai–Tibet Plateau sampling sites at three altitudes.

Tibetan sheep of plateau type (Euler type, 3.5 years old, 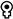, n = 6/group, Non-pregnant) were selected as research subjects. All sheep were under traditional local natural grazing management and no supplementary feeding was carried out, and grazed on fixed pastures from 8 am to 7 pm. In August 2020, samples were collected in Zhuoni (LA, 2500 m, Gansu province), Haiyan (MA, 3500 m, Qinghai province), and Yushu (HA, 4500 m, Qinghai province) in the Qinghai–Tibet Plateau region (Figure 12), and the three sampling sites belong to Qinghai Plateau and its adjacent areas.

### 4.2. Sample Collection

The method reported by Ma et al. [64] was used to collect plateau forage samples from 10 plots (50 cm × 50 cm), and the distance between plots is more than 10 m. The dominant forage species at each altitude were analyzed, and the height of the dominant species was determined using a ruler. Subsequently, the aboveground biomass was collected and brought back to the laboratory for drying in an oven at 65 °C for 48 h. The initial moisture was determined once the sample reached a constant weight. The forage sample was crushed with a mill equipped with a 1 mm sieve, after which chemical composition was determined.

Animal samples were collected in accordance with ethics committee approval (Approval No. GAU-Eth-AST-2021-001). All sheep were taken to the abattoir for sampling and fasted 24 h before euthanasia. Before the euthanasia, jugular vein blood was collected in a vacuum tube (without anticoagulant), and then left for about half an hour before centrifugation (5000× *g*, 20 min, and 4 °C). The serum was separated and stored at −20 °C to be used later in the determination of immune indices. Rumens were removed within 10 min after slaughter, and rumen epithelial tissues (abdominal sacs) were collected and washed with normal saline. Part of the samples were put into cryogenic vials for rapid freezing for subsequent transcriptome sequencing, and the other part was removed and fixed in 4% paraformaldehyde for morphological analysis. Then, about 50 mL of rumen contents (per sheep) were collected, divided into cryogenic vials, quickly frozen in a liquid nitrogen tank, and used to determine 16S rRNA and VFAs.

### 4.3. Determination of Forage Nutrient Composition and Rumen VFAs

The dry matter (DM), crude protein (CP), ether extract (EE), calcium (Ca), and phosphorus (P) of forage grasses were determined according to the AOAC method [65], and the content of acid detergent fiber (ADF) and neutral detergent fiber (NDF) was determined according to the Van Soest method [66]. The composition and content of VFAs were determined by gas chromatograph (GC-2010 plus), and the specific method was referred to the study of Liu et al. [13].

### 4.4. Serum Immune Index Determination

The serum concentrations of immunoglobulin A (IgA), immunoglobulin M (IgM), and immunoglobulin G (IgG) were determined using the enzyme-linked immunosorbent assay method (ELISA, Nanjing Jiancheng Institute of Bioengineering Ltd., Nanjing, China), and the assay was performed with a Microplate Reader (Thermo Fisher Scientific, Waltham, MA, USA). The coefficient of variation of the ELISA kit was less than 20%, and the detection range of IgA, IgM, and IgG kits was 0.8–24 μg/mL, 50–1500 μg/mL, and 0.2–18 μg/mL, respectively.

### 4.5. Morphological Analysis

The collected abdominal sac tissues of the rumen were rinsed with normal saline, trimmed into 1 cm × 1 cm tissue blocks, and fixed in 4% paraformaldehyde for 24 h, and then dehydrated, cleared, waxed, embedded, sliced, and dyed. Tissue sections were stained with hematoxylin and eosin, which stained the nucleus blue-purple and the cytoplasm pink. The morphology of the rumen at different altitudes was observed and compared under a light microscope. Five HE-stained sections were taken from each rumen, and five fields of view were selected for each section and photographed digitally. The thickness of each layer of the rumen epithelium and the width and length of the papilla were measured and analyzed by CaseViewer slice analysis system.

### 4.6. DNA Extraction and 16S rRNA Sequencing

Rumen microbial DNA was extracted by MN NucleoSpin 96 Soil kit (Macherey-Nagel, Düren, Germany), and the specific experimental procedures were carried out according to the kit guidelines. The concentration and purity of DNA were detected by NanoPhotometer (N60, Germany) to ensure the integrity of the extracted DNA samples for subsequent sequencing. The V3–V4 region of 16S rRNA gene was amplified by PCR to further analyze the community structure of rumen microbiota. The primers were (338F 5′-ACTCCTACGGGAGGCAGCAG-3′ and 806R 5′-GGACTACHVGGGTWTCTAAT-3′). Sequencing was performed on the Illumina MiSeq platform (Illumina, San Diego, CA, USA), and the bioinformatics analysis was performed using BMKCloud (www.biocloud.net (accessed on 22 November 2020)). To evaluate the quality of the raw data coming back from sequencing, and paired-end splicing (FLASH v1.2.7) and filtering (Trimmomatic v0.33), chimeras (UCHIME v4.2) were removed to obtain optimized sequences (Tags). The OTU was obtained using the Usearch software [67]; OTU classification and annotation were analyzed by using Silva (Bacteria) taxonomy database for further taxonomic analysis, and the community structure at different taxonomic levels (phylum, class, order, family, genus, and species) were obtained; the species diversity was analyzed through Alpha diversity, and the Alpha diversity index Ace, Chao1, Shannon, and Simpson were obtained, and a sample rarefaction curve was drawn [68]. Furthermore, Beta diversity analysis was used to obtain PCoA [69] and boxplots based on multiple distances according to the distance matrix (Anosim). LEfSe analysis was used to find Biomarkers with statistical differences between groups [70]; the software Metastats was used to conduct *t*-tests on species abundance data between groups [71]; according to the q value, the species that cause the difference in the composition of the two groups of samples are screened out; 16S gene function analysis was performed through KEGG and COG [72].

### 4.7. RNA Extraction and Transcriptome Sequencing

Total RNA was extracted from rumen epithelial tissues of Tibetan sheep by Trizol reagent kit (Invitrogen, Carlsbad, CA, USA), and specific experimental procedures were carried out according to the kit guide, and all extraction processes were carried out on the ultra-clean table. The concentration and purity were detected by ultra-micro spectrophotometer (Therm Nano Drop-2000), and RNA concentration (ng/uL) and purity (260 nm/280 nm ratio (1.8–2.1) were recorded respectively. The cDNA Library was constructed by the VAHTS Universal V6 RNA-SEQ Library Prep Kit for Illumina^®^ Kit (NR604-02); refer to the kit guide for specific steps. The cDNA product was further purified (VAHTSTM DNA Clean Beads kit, N411–03). Quality inspection, library construction, and then Illumina NovaseQ6000 (San Diego) platform sequencing were performed. Clean data were obtained by filtering the off-machine data. Mapped Data from sequence alignment with the specified reference genome Ovis_aries (Oar_rambouillet_v1.0. Ovis_aries) were obtained by HISAT [73]. StringTie [74] was used to compare reads on the pair for assembly. Then, a bioinformatics analysis was performed on the Biomark cloud platform BMKCloud (www.biocloud.net (accessed on 22 November 2020)). FPKM [75] (fragments per kilobase of transcript per million fragments mapped) was used to measure the gene expression. A Fold change >= 2 and FDR < 0.01 was used as a standard for differential gene screening, and the Gene Ontology (GO) and Kyoto Encyclopedia of Genes and Genomes (KEGG) functional enrichment analyses of differential genes were performed by GOseq [76].

### 4.8. Data Analysis

Excel 2016 was used to sort out the data, and IBM SPSS Statistics (V.22.0) was used to analyze the significance of forage chemical composition, serum immune indexes, rumen morphological data, and VFAs. Single-factor ANOVA analysis and Duncan’s multiple test were performed, *p* < 0.05 was considered a significant difference, *p* < 0.01 was considered a very significant difference, and the results were expressed as the Mean value and Standard Error of Mean (SEM). Weighted gene co-expression network analysis (WGCNA) [77] was performed to understand the link between the host transcriptome (mRNA) and the Tibetan sheep phenotypic traits (VFAs and morphological indexes). The Spearman correlation test was performed to analyze the correlation between genus-level microbial species (Top20) and module gene expression.

## 5. Conclusions

We demonstrate that Tibetan sheep respond to plateau environmental pressure through rumen microbial–host gene interaction. With the increase in altitude, the protein content and trace element content of plateau forage increased, leading to the improvement in rumen fermentation performance. Among them, the content of fermentation metabolites VFAs decreased in Tibetan sheep at high altitude (HA), and the ratio of acetic acid to propionic acid (A/P) was significantly decreased, which improved the energy utilization of forage. The morphological structure of the rumen epithelium also changed accordingly, with increased absorption area and epithelial permeability, and the immune levels of IgA and IgG increased with altitude. Under the environmental pressure at different altitudes, the rumen microbial diversity of Tibetan sheep gradually decreased with the increase in altitude. Rumen microbes and rumen epithelium genes interact in Tibetan sheep at high altitude, and are co-enriched in the pathways of “Energy production and conversion”, “Lipid transport and metabolism”, and “Defense mechanisms”. They synergistically participate in rumen fermentation metabolism and epithelial immune barrier function, so as to improve the metabolism and immune level of Tibetan sheep at high altitude.

## Figures and Tables

**Table 1:** Nutrient composition of forage at different altitudes (DM basis)/%.

	LA	MA	HA	SEM	*p*-Value
CP	11.25 ^b^	10.06 ^c^	17.81 ^a^	0.91	<0.01
EE	4.29 ^a^	3.77 ^b^	4.18 ^b^	0.09	0.03
Ash	7.34 ^b^	4.55 ^c^	9.18 ^a^	2.18	<0.01
NDF	58.08 ^b^	70.11 ^a^	50.40 ^c^	0.91	<0.01
ADF	33.98 ^b^	36.17 ^a^	28.14 ^c^	1.39	<0.01
HCEL	24.10 ^b^	33.94 ^a^	22.26 ^c^	0.14	<0.01
NFC	19.02 ^a^	11.50 ^b^	18.43 ^a^	0.95	<0.00
Ca	0.89 ^b^	0.65 ^b^	1.80 ^a^	0.10	<0.01
P	1.21 ^a^	0.70 ^b^	1.52 ^a^	0.91	<0.01

Note: Different superscript lowercase letters indicate significant differences on the same line, *p* < 0.05; CP: Crude protein; EE: Crude fat; DM: Dry matter; Ash: Crude ash; NDF: Neutral detergent fiber; ADF: Acid detergent fiber; HCEL: Hemicellulose; NFC: Non-fiber carbohydrates.

**Table 2:** Rumen VFAs of Tibetan sheep at different altitudes.

	LA	MA	HA	SEM	*p*-Value
Acetic Acid (mmol/L)	53.59b ^b^	71.06 ^a^	36.21 ^c^	3.76	<0.00
Propanoic Acid (mmol/L)	7.64 ^b^	11.75 ^a^	7.05 ^b^	0.68	0.00
Isobutyric acid (mmol/L)	1.47 ^a^	1.53 ^a^	0.73 ^b^	0.09	<0.00
Butyric acid (mmol/L)	7.92 ^a^	5.89 ^b^	3.37 ^c^	0.53	<0.00
Isovaleric acid (mmol/L)	1.94 ^a^	1.81 ^a^	0.84 ^b^	0.12	<0.00
Valeric Acid (mmol/L)	0.25	0.28	0.52	0.08	0.35
Total-VFAs (mmol/L)	72.79 ^b^	92.32 ^a^	48.72 ^c^	4.79	<0.00
A/P	7.03 ^a^	6.22 ^b c^	5.37 ^c^	0.28	0.04

Note: Different superscript lowercase letters indicate significant differences on the same line, *p* < 0.05; A/P: Acetic Acid/Propanoic Acid.

**Table 3:** Microbial diversity indices at different altitudes.

Index	LA	MA	HA	SEM	*p*-Value
Shannon	8.33 ^a^	7.62 ^b^	7.62 ^b^	0.10	0.00
Simpson	0.99	0.99	0.98	0.00	0.25
ACE	1325.88 ^a^	1317.05 ^a^	1241.72 ^b^	15.58	0.04
Chao1	1346.56	1355.22	1281.25	15.02	0.08
PD_whole_tree	69.27	67.39	67.04	0.63	0.31
Coverage	0.99695	0.995867	0.996917	0.00	0.10

Note: Different superscript lowercase letters indicate significant differences on the same line, *p* < 0.05.

## Data Availability

The datasets presented in this study can be found in online repositories. The names of the repository/repositories and accession numbers can be found below: (Sequence Read Archive (SRA): PRJNA818841 (Microbial sequence)/PRJNA819418 (Transcriptome sequence)).

## Supplementary Materials

The following supporting information can be downloaded at: https://www.mdpi.com/article/10.3390/ijms232012430/s1.
