# Supplementary material for: Response of Ruminal Microbiota–Host Gene Interaction to High-Altitude Environments in Tibetan Sheep
Source: Int J Mol Sci. 2022 Oct 17;23(20):12430. doi: 10.3390/ijms232012430 (PMC9604387; doi:10.3390/ijms232012430)
Supplement: Supplementary file 1 [file ijms-23-12430-s001.zip › ijms-1960878-supplementary/Table S1.pdf]

Table S1. Statistics of rumen epithelial structure of Tibetan sheep at different altitudes

	LA	MA	HA
Muscular layer	1381.18±85.88^{c}	2067.68±133.51^{a}	1868.96±86.66^{b}
Length of the nipple	1916.60±193.07^{ab}	2020.96±215.66^{a}	1720.36±117.10^{b}
Width of the nipple	463.92±25.69^{b}	403.36±13.73^{c}	504.58±20.50^{a}
SC	34.40±2.92^{b}	43.42±2.07^{a}	36.20±2.69^{b}
SG	21.46±2.39^{a}	23.44±1.27^{a}	16.12±0.95^{b}
SS	34.90±2.77^{b}	35.26±2.73^{b}	43.76±1.35^{a}
BL	47.52±2.97^{b}	41.26±2.61^{c}	63.26±4.66^{a}

Note: Different superscript lowercase letters indicate significant differences on the same line, $P<0.05$; the same or no letters mean the difference is not significant, $P>0.05$; SC: Stratum corneum; SG: Stratum granulosum; SS: Stratum spinosum; BL: Basal layer
