# Supplementary figures and images for: Response of Ruminal Microbiota–Host Gene Interaction to High-Altitude Environments in Tibetan Sheep
Source: Int J Mol Sci. 2022 Oct 17;23(20):12430. doi: 10.3390/ijms232012430 (PMC9604387; doi:10.3390/ijms232012430)

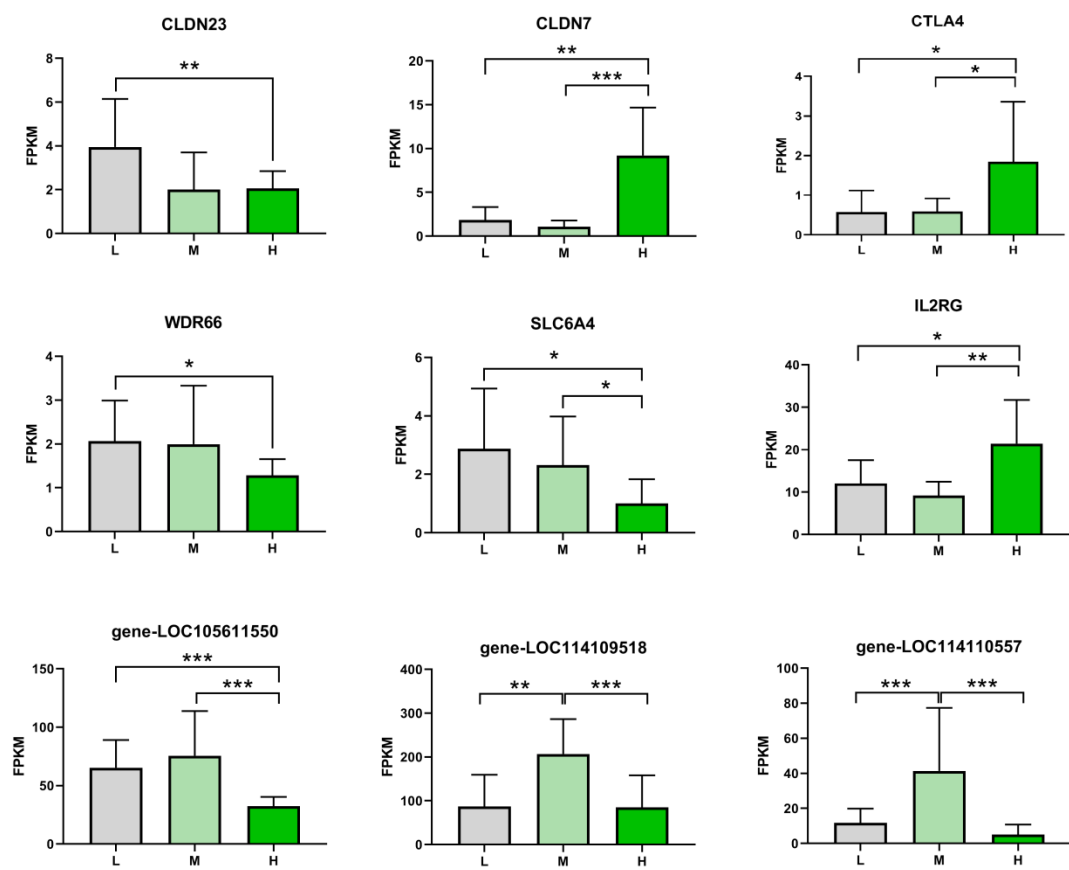

**Figure S1. Partial module gene expression**

\* $P < 0.05$ , \*\* $P < 0.01$ , \*\*\* $P < 0.001$ .

Supplement: Supplementary file 1 [file ijms-23-12430-s001.zip › ijms-1960878-supplementary/Figure S1.pdf]

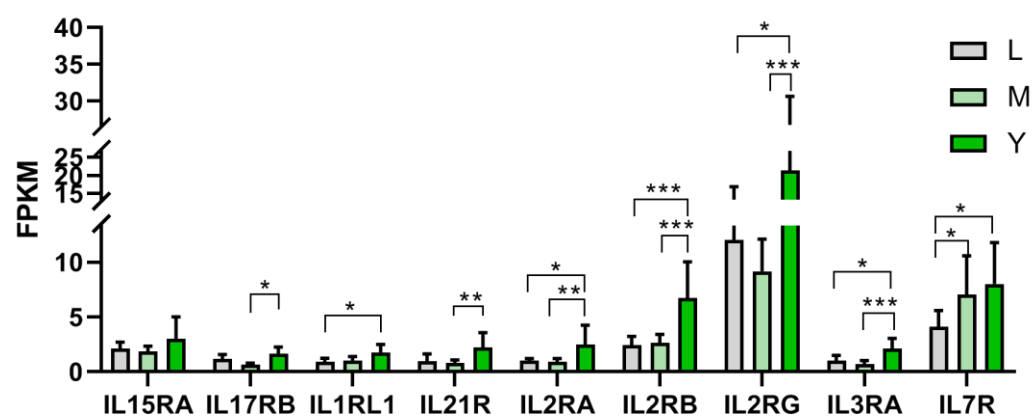

**Figure S2.** IL family gene expression

\* $P < 0.05$ , \*\* $P < 0.01$ , \*\*\* $P < 0.001$ .

Supplement: Supplementary file 1 [file ijms-23-12430-s001.zip › ijms-1960878-supplementary/Figure S2.pdf]
